# Risk factors and outcomes of intraoperative hypothermia in neonatal and infant patients undergoing general anesthesia and surgery

**DOI:** 10.3389/fped.2023.1113627

**Published:** 2023-03-15

**Authors:** Jialian Zhao, Zhenkai Le, Lihua Chu, Yi Gao, Manqing Zhang, Jiabin Fan, Daqing Ma, Yaoqin Hu, Dengming Lai

**Affiliations:** ^1^ Department of Anesthesiology, Children's Hospital, Zhejiang University School of Medicine, National Clinical Research Center for Child Health, Hangzhou, China; ^2^ Department of Neonatal Surgery, Children's Hospital, Zhejiang University School of Medicine, National Clinical Research Center for Child Health, Hangzhou, China; ^3^ Department of Anesthesiology, First Affiliated Hospital, Zhejiang University School of Medicine, Hangzhou, China; ^4^ Division of Anaesthetics, Pain Medicine & Intensive Care, Department of Surgery & Cancer, Faculty of Medicine, Imperial College London, Chelsea & Westminster Hospital, London, United Kingdom

**Keywords:** perioperative hypothermia, neonate, infant, pre-warming, thermal management, intraoperative hypothermia

## Abstract

**Objective:**

The incidence of intraoperative hypothermia remains high in pediatric patients during anesthesia and surgery even though core body temperature monitoring and warming systems have been greatly improved in recent years. We analyzed the risk factors and outcomes of intraoperative hypothermia in neonates and infants undergoing general anesthesia and surgery.

**Methods:**

The data on the incidence of intraoperative hypothermia, other clinical characteristics, and outcomes from electronic records of 1,091 patients (501 neonates and 590 infants between 28 days and 1 year old), who received general anesthesia and surgery, were harvested and analyzed. Intraoperative hypothermia was defined as a core temperature below 36°C during surgery.

**Results:**

The incidence of intraoperative hypothermia in neonates was 82.83%, which was extremely higher than in infants (38.31%, *p* < 0.001)—the same as the lowest body temperature (35.05 ± 0.69°C vs. 35.40 ± 0.68°C, *p* < 0.001) and the hypothermia duration (86.6 ± 44.5 min vs. 75.0 ± 52.4 min, *p* < 0.001). Intraoperative hypothermia was associated with prolonged PACU, ICU, hospital stay, postoperative bleeding, and transfusion in either age group. Intraoperative hypothermia in infants was also related to prolonged postoperative extubation time and surgical site infection. After univariate and multivariate analyses, the age (OR = 0.902, *p* < 0.001), weight (OR = 0.480, *p* = 0.013), prematurity (OR = 2.793, *p* = 0.036), surgery time of more than 60 min (OR = 3.743, *p* < 0.001), prewarming (OR = 0.081, *p* < 0.001), received >20 mL/kg fluid (OR = 2.938, *p* = 0.004), and emergency surgery (OR = 2.142, *p* = 0.019) were associated with hypothermia in neonates. Similar to neonates, age (OR = 0.991, *p* < 0.001), weight (OR = 0.783, *p* = 0.019), surgery time >60 min (OR = 2.140, *p* = 0.017), pre-warming (OR = 0.017, *p* < 0.001), and receive >20 mL/kg fluid (OR = 3.074, *p* = 0.001) were relevant factors to intraoperative hypothermia in infants along with the ASA grade (OR = 4.135, *p* < 0.001).

**Conclusion:**

The incidence of intraoperative hypothermia was still high, especially in neonates, with a few detrimental complications. Neonates and infants each have their different risk factors associated with intraoperative hypothermia, but younger age, lower weight, longer surgery time, received more fluid, and no prewarming management were the common risk factors.

## Introduction

Intraoperative hypothermia, generally defined as a decrease in core temperature below 36°C during surgeries, is one of the most common physiological disturbances during general anesthesia in pediatric patients (1, 2). Hypothermia is closely related to postoperative cardiovascular events (3), coagulopathies (4), surgical-wound infection (5), nausea and vomiting (6), pain (7), increased blood loss, and prolonged recovery time (8, 9). In neonates and infants, cold stress can induce multiple pathophysiological distress such as catecholaminergic response, vasoconstriction, increased metabolism, and decreased lung surfactant synthesis; all these may lead to pulmonary hypertension, tissue hypoxia, arterial hypotension, hypoperfusion metabolic acidosis, and hypoglycaemia (10).

Anesthesia induction can cause body temperature to decrease by 1°C–2°C, especially in young children (11), because of the lower body weight, relatively large body surface, and immature thermoregulatory system (12). Although core body temperature monitoring and active and passive temperature management were implemented to reduce intraoperative hypothermia incidence, it was still reported to be as high as 50% in children (13, 14) and 85% in neonates (15). The current study was to retrospectively investigate the risk factors and outcomes of intraoperative hypothermia in neonates and infants undergoing general anesthesia and surgery.

## Methods

This retrospective study was conducted at the Children's Hospital, Zhejiang University School of Medicine, Hangzhou, China. The study protocol was approved by the Ethics Committee (2018-IRB-048) and registered in the Chinese Clinical Trial Registry (ChiCTR1800018863; principal investigator: Jialian Zhao; date of registration: 14 October 2018). Neonates and infants receiving general anesthesia for more than 30 min were enrolled. The study recorded perioperative data of consecutive infants (between 1 and 12 months old) from 1 October 2021 to 31 March 2022 and neonates from 1 April 2021 to 31 March 2022 who underwent noncardiac surgeries. Exclusion criteria included patients with (1) preoperative hypothermia (<36°C) or hyperthermia (>38°C); (2) core temperature >38.5°C due to infection or other reasons within three days before surgery; (3) a history of hypothyroidism or hyperthyroidism or other endocrine disorders which may influence body temperature; (4) thermoregulation abnormalities such as malignant hyperthermia or neuroleptic malignant syndrome; (5) therapeutic hypothermia; (6) preoperative drugs usage that might influence body temperature such as NSAIDs; and (7) no core temperature monitored or data lost.

All patients received intravenous anesthesia (propofol- and remifentanil-based) or sevoflurane-based inhalational general anesthesia or combined. Core temperatures were monitored *via* an esophageal or nasopharyngeal temperature probe (Opper, China) after intubation and throughout the surgery. All patients received passive and active warming during the surgery, and several patients received active prewarming. Active prewarming was defined as patients starting receiving active warming with a Forced-Air Warmer (Covidien llc, United States) and a blanket before induction of anesthesia and lasted the whole surgical process according to the patients’ body temperature. In addition, all patients received warming infusion solution and forced air warming on and off during the surgery as necessary. Once the core temperature fell below 36°C in the electronic system, the patient was defined as having hypothermia. The lowest temperature and duration of hypothermia were also harvested. If the hypothermia lasts more than 24 h, it is defined as persistent hypothermia. However, due to the incompletion of data recording, how long and how often these active warming systems were being used was not found in the recording system. The accurate temperature of the operating room during the surgery was also not found.

Patients’ demographics and intraoperative parameters such as age, gender, weight, gestational age of the neonate, ASA (American Society of Anesthesiologists physical status classification system) grade, type and site of surgery, duration of anesthesia and surgery, artery catheterization/central vena catheterization (CVC) use, infusion, blood transfusion, vasoactive drugs use, blood loss, and urine volume were harvested. The postoperative extubation time, length of PACU stay, postoperative ICU admission, mechanical ventilation in ICU, length of ICU and postoperative hospital stay, mortality within postoperative 30 days, and postoperative complications such as surgical site infection (SSI), deep venous thrombosis (DVT), postoperative bleeding, and transfusion were also obtained.

### Statistical analysis

Quantitative characteristics such as age and weight were presented as mean ± SD or median (IQR) and compared by Student’s *t*-test or the Mann–Whitney test as appropriate, while categorical variables were presented as *n* (%) and analyzed with the chi-Square test. Variables that were significant in univariate analysis were subjected to binary logistic regression (Forward, LR, an entry level of 0.05, and an exclusion level of 0.1) for the determination of risk factors. All statistical tests were two-sided and performed by SPSS 20.0 software. A *p*-value <.05 was considered statistically significant.

## Results

### Hypothermia and its associated outcomes

**Figure 1:**
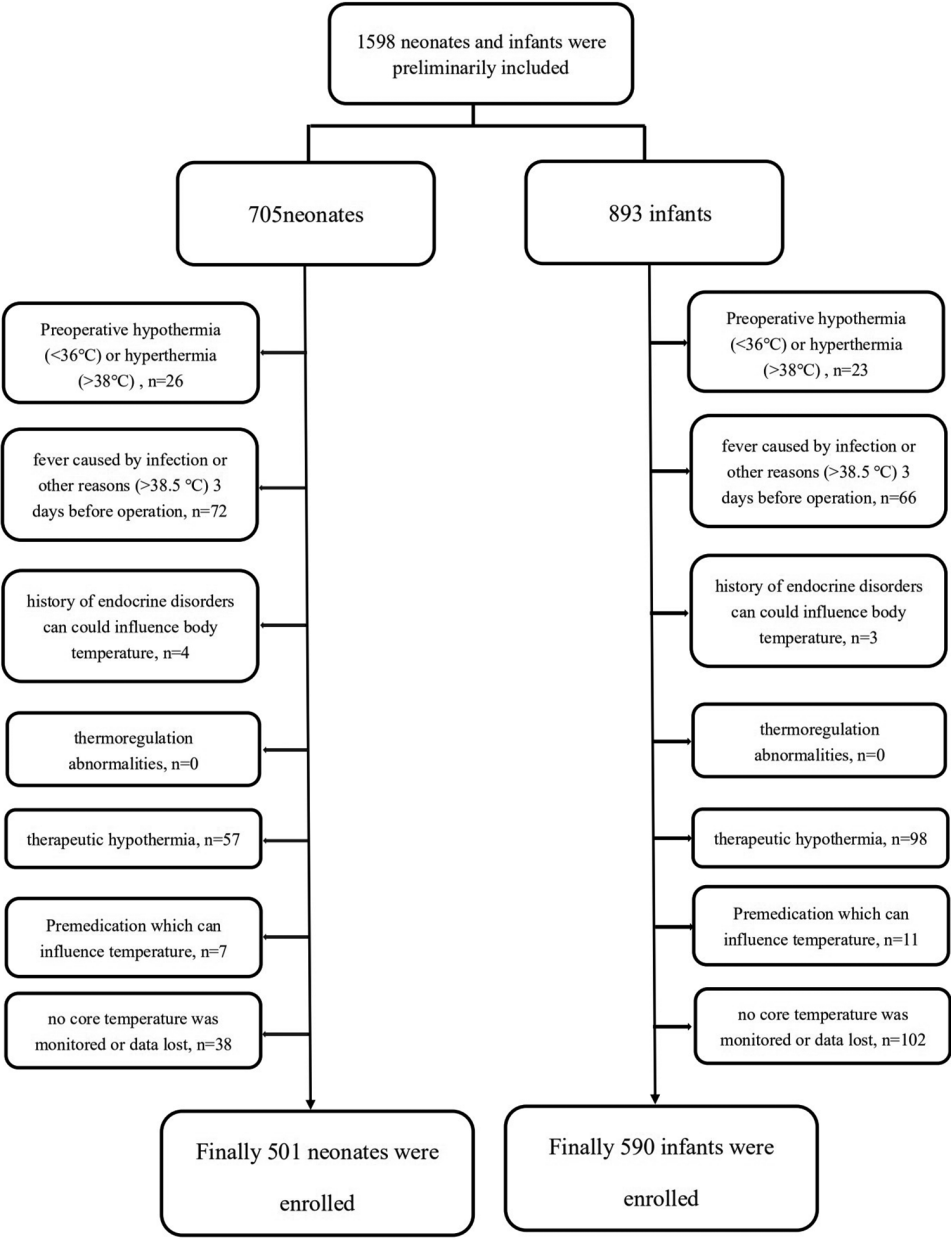
Flow chart of inclusion and exclusion criteria of neonates and infants.

**Figure 2:**
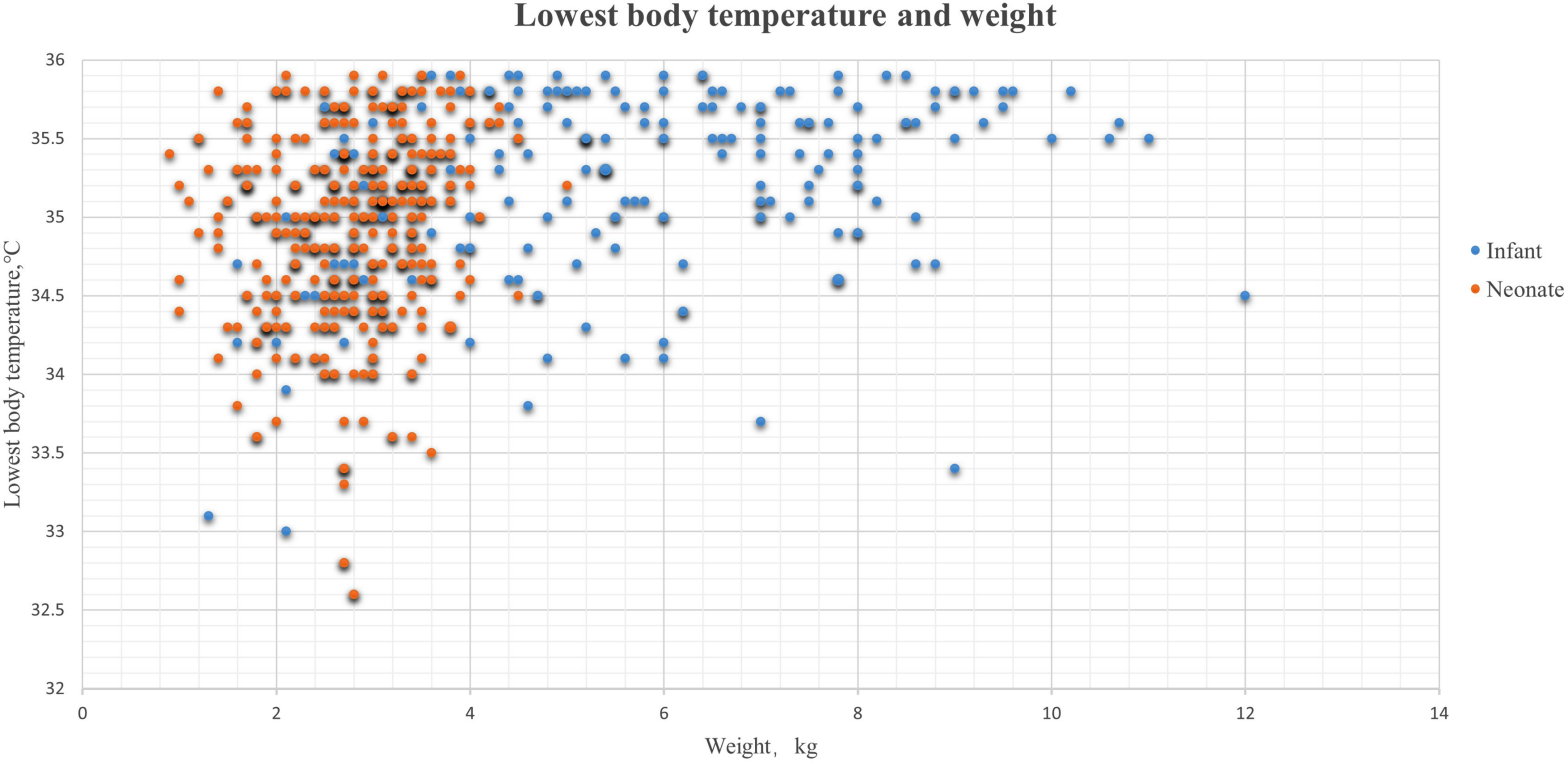
Lowest body temperature and weight in hypothermia patients. The blue dots were the lowest body temperatures during the surgeries in infant patients, and the orange dots represent the lowest body temperature in neonates.

A total of 705 consecutive neonates and 893 infants (between 28 days and 12 months old) were enrolled in this retrospective study. After exclusion, 501 neonates and 590 infants were included (Figure 1). The overall incidence of hypothermia was 82.83% in neonates, which was extremely higher than 38.31% in infant patients (*p* < .001). Hypothermia tended to be much more severe in neonates (Figure 2). Among these, the lowest body temperature in neonates was 35.05 ± 0.69°C and lasted for 86.6 ± 44.5 min, while it was 35.40 ± 0.68°C in infants and lasted for 75.0 ± 52.4 min. All these were more significant in neonates than infants (*p* < .001 and *p* = .004, respectively). Patients were classified into four levels according to their lowest body temperature; most patients (55.67% in neonates and 77.43% in infants) had mild hypothermia between 35.0°C and 35.9°C. Neonates had more severe (33.0°C–33.9°C) or extremely severe (<33°C) hypothermia, while none of the infants had extremely severe hypothermia. Neonates were also more likely to have persistent hypothermia than infants after surgeries (2.17% vs. 1.32%, *p* = .042). Prewarming management with a forced-air warming system was used in 59.88% of neonates, which was significantly higher than in infants (31.36%, *p* < .001) (Table 1).

**Table 1:** Characteristics of hypothermia in neonates and infants.

	Neonate (*n *= 501)	Infant (*n *= 590)	*p*
Overall incidence of hypothermia, %	415 (82.83%)	226 (38.31%)	<.001
Lowest body temperature, °C	35.05 ± 0.69	35.40 ± 0.68	<.001
Level of hypothermia, *n* (%)			<.001
Mild 35°C–35.9°C	231 (55.67%)	175 (77.43%)	
Moderate 34°C–34.9°C	165 (39.96%)	44 (19.47%)	
Severe 33°C–33.9°C	15 (3.61%)	7 (3.10%)	
Extremely severe <33	4 (0.96)	0	
Time of hypothermia (min)	86.6 ± 44.5	75.0 ± 52.4	.004
Prewarming, *n* (%)	300 (59.88%)	185 (31.36%)	<.001
Persistent hypothermia, *n* (%)	9 (2.17%)	3 (1.32%)	.042

Intraoperative hypothermia led to a longer PACU stay, more postoperative ICU admission, longer ICU stay, longer postoperative mechanical ventilation time, and longer postoperative hospital stay in neonates than in infants (all *p* values <.05). Hypothermia also led to a longer postoperative extubation time in the PACU in infants (18.3 ± 16.2 min vs.14.0 ± 9.9 min, *p* = .017). Surgical site infection (SSI) was more frequent in hypothermia patients, and there was a significant difference in the incidence of infection in infants with hypothermia than in those without hypothermia (2.65% vs. 0.27%, *p* = .014). Furthermore, intraoperative hypothermia resulted in a higher risk of postoperative bleeding (17.11% vs. 8.13%, *p* = .037 in neonates; 5.8% vs. 1.11%, *p* = .002 in infants) and postoperative transfusion in ICU (26.27% vs. 12.80%, *p* = .008 in neonates and 26.11% vs. 6.04%, *p* < .001 in infants). There was no significant difference in mortality within postoperative 30 days, even though the mortality was slightly higher in patients with hypothermia patients (Table 2).

**Table 2:** Outcomes of hypothermia and normothermia.

	Neonate (*n *= 501)			Infant (*n *= 590)		
	Hypothermia (*n *= 415)	Normothermia (*n *= 86)	*p*-Value	Hypothermia (*n *= 226)	Normothermia (*n *= 364)	*p*-Value
Postoperative extubation time in PACU (min)	31.3 ± 27.8	14.5 ± 6.11	0.366	18.3 ± 16.2	14.0 ± 9.9	.017
Length of PACU stay (min)	59.3 ± 4.6	41.3 ± 12.6	0.067	56.7 ± 24.1	48.0 ± 15.6	.003
Postoperative ICU admit, *n* (%)	412 (99.28%)	82 (95.35%)	0.020	178 (78.76%)	189 (51.92%)	<.001
Time of mechanical ventilation (h)	7 (5–12)	5 (4–8)	<0.001	3 (2–4)	2.5 (2–3)	<.001
Length of ICU stay (days)	2 (1–9)	1 (1–3)	<0.001	1 (1–2)	1 (1–1)	<.001
Length of postoperative hospital stay (days)	14 (8–24)	11 (7–17)	0.003	7 (5–15)	5 (2.25–8)	<.001
Surgical site infection (SSI)	12 (2.89%)	1 (1.16%)	0.707	6 (2.65%)	1 (0.27%)	.014
Postoperative pneumonia, *n* (%)	8 (1.93%)	2 (2.33%)	0.810	7 (3.10%)	11 (3.02%)	.959
Deep venous thrombosis (DVT), *n* (%)	5 (1.20%)	0 (0%)	0.594	4 (1.77%)	5 (1.37%)	.703
Postoperative bleeding, *n* (%)	71 (17.11%)	7 (8.13%)	0.037	13 (5.80%)	4 (1.11%)	.002
Transfusion in ICU, *n* (%)	109 (26.27%)	11 (12.80%)	0.008	59 (26.11%)	22 (6.04%)	<.001
Mortality within postoperative 30 days, *n* (%)	15 (3.61%)	1 (1.12%)	0.329	4 (1.77%)	2 (0.55%)	.310

PACU, postanesthesia care unit; ICU, intensive care unit; SSI, surgical site infection; DVT, deep venous thrombosis.

### Demographics, perioperative parameters, hypothermia, and univariate analysis

Patients with hypothermia were much younger (7.3 ± 6.9 days vs. 11.7 ± 8.0 days, *p* < .001 in neonates and 120.4 ± 83.4 days vs. 204.7 ± 103.3 days, *p* < .001, in infants) and had lower weight (2.8 ± 0.7 kg vs. 3.2 ± 0.6 kg, *p* < .001 in neonates and 5.9 ± 2.2 kg, vs. 7.7 ± 2.1 kg, *p* < .001 in infants). The ASA grade was higher in hypothermia patients (*p* < .001). Preterm neonates or infants, who were preterm, were more common in the hypothermia group (33.77% vs. 12.79%, *p* < .001 in neonates, while 26.54% vs. 16.76%, *p* = .004 in infants). Patients with hypothermia had a longer surgery time both in neonates and infants (86.5 ± 39.6 vs. 57.8 ± 38.5 min, *p* < .001 in neonates; 109.0 ± 72.2 vs. 68.8 ± 54.8 min, *p* < .001 in infants). Major surgeries with longer anesthesia time and more invasive anesthesia procedures, including artery catheterization and central vena catheterization, were higher in the hypothermia group (all *p*’s < .05). The use of prewarming with a forced-air warming system was significantly lower in hypothermia incidence (55.67% vs. 80.23% *p* < .001 in neonates; 14.16% vs. 42.03%, *p* < .001 in infants). Hypothermal neonates and infants received more perioperative fluid, blood transfusion, and vasoactive drugs and had more blood loss during surgeries (all *p*’s < .05) (Table 3).

**Table 3:** Basic characteristics and univariate analysis results of hypothermia in neonates and infants.

	Neonate (*n *= 501)			Infant (*n *= 590)		
	Hypothermia (*n *= 415)	Normothermia (*n *= 86)	*p*-Value	Hypothermia (*n *= 226)	Normothermia (*n *= 364)	*p*-Value
Age (day)	7.3 ± 6.9	11.7 ± 8.0	<0.001	120.4 ± 83.4	204.7 ± 103.3	<.001
Gender (male)	229 (55.18%)	46 (53.5%)	0.774	129 (57.08%)	205 (56.32%)	.885
Body weight (kg)	2.8 ± 0.7	3.2 ± 0.6	<0.001	5.9 ± 2.2	7.7 ± 2.1	<.001
ASA classification, *n* (%)			<0.001			<.001
I	1 (0.24%)	2 (2.33%)		33 (14.60%)	169 (46.43%)	
II	267 (64.34%)	72 (83.73%)		135 (59.73%)	190 (52.20%)	
III	128 (30.84%)	10 (11.63%)		48 (21.24%)	2 (0.55%)	
≥IV	19 (4.58%)	2 (2.33%)		9 (3.98%)	3 (0.82%)	
Prematurity, *n* (%)	136 (33.77%)	11 (12.79%)	<0.001	60 (26.54%)	61 (16.76%)	.004
Correct gestational age (week)	38.09 ± 2.83	40.02 ± 2.46	<0.001	54.14 ± 13.01	67.03 ± 15.09	<.001
Emergency surgery, *n* (%)	339 (81.68%)	55 (63.95%)	<0.001	54 (23.89%)	59 (16.21%)	.021
Type of surgery, *n* (%)			0.002			<.001
General surgery	336 (80.96%)	63 (73.26%)		134 (59.29%)	150 (41.21%)	
Thoracic surgery	44 (10.60%)	8 (9.30%)		19 (8.41%)	28 (7.69%)	
Orthopedics surgery	0	2 (2.33%)		1 (0.44%)	2 (0.55%)	
Neurosurgery	30 (7.23%)	11 (12.79%)		35 (15.49%)	76 (20.88%)	
Urology	0	0		15 (6.64%)	6 (1.65%)	
Plastic surgery	0	0		13 (5.75%)	52 (14.29%)	
ENT	1 (0.24%)	2 (2.33%)		3 (1.33%)	36 (9.89%)	
Other	4 (0.96%)	0		6 (2.65%)	14 (3.85%)	
Site of surgery, *n* (%)			0.035			<.001
Head and neck	31 (7.47%)	13 (15.12%)		43 (19.02%)	104 (28.57%)	
Thorax and dorsum	34 (8.19%)	8 (9.30%)		37 (16.37%)	96 (26.37%)	
Abdomen	338 (81.45%)	64 (74.42%)		143 (63.27%)	149 (40.93%)	
Limbs	0	1 (1.16%)		2 (0.88%)	6 (1.65%)	
Others	1 (0.24%)	0		1 (0.44%)	9 (2.47%)	
Invasiveness of surgery, *n* (%)			0.037			.021
Endoscopic surgery	110 (26.51%)	33 (38.37%)		101 (44.69%)	128 (35.16%)	
Open surgery	305 (73.49%)	53 (61.63%)		125 (55.31%)	238 (65.38%)	
Time of surgery (min)	86.5 ± 39.6	57.8 ± 38.5	<0.001	109.0 ± 72.2	68.8 ± 54.8	<.001
Time of anesthesia (min)	138.1 ± 45.8	102.6 ± 48.4	<0.001	163.0 ± 77.1	114.7 ± 60.9	<.001
Artery catheterization, *n* (%)	376 (90.60%)	56 (65.12%)	<0.001	174 (76.99%)	154 (42.31%)	<.001
Central vena catheterization (CVC), *n* (%)	186 (44.82%)	28 (32.56%)	0.036	96 (42.48%)	71 (19.51%)	<.001
Prewarming, *n* (%)	231 (55.67%)	69 (80.23%)	<0.001	32 (14.16%)	153 (42.03%)	<.001
Perioperative IV fluid (mL/kg)	30.07 ± 16.02	18.90 ± 12.21	<0.001	38.90 ± 39.00	20.91 ± 16.70	<.001
Blood transfusion, *n* (%)	82 (19.76%)	8 (9.30%)	0.022	41 (18.14%)	18 (4.95%)	<.001
Vasoactive drugs, *n* (%)	64 (15.42%)	6 (6.98%)	0.040	19 (8.41%)	7 (1.92%)	<.001
Blood loss, mL/kg	2.30 ± 2.70	1.22 ± 1.65	0.01	2.48 ± 9.33	0.66 ± 1.55	<.001
Urine, mL/kg	5.02 ± 4.63	4.43 ± 5.01	0.432	12.23 ± 17.63	7.33 ± 7.00	.001

ASA, American Society of Aneshesiologists physical status classification system; ENT, ear, nose, and throat; CVC, central vena catheterization; IV, infusion of the vein.

### Factors associated with hypothermia

Younger age, lower weight, longer surgery time, received more fluid, and no prewarming were common risk factors for hypothermia in neonates and infants (Table 4). Preterm and emergency surgery also showed a strong association with hypothermia in neonates, while higher ASA grade was a risk factor in infants. The risk factors for hypothermia in neonates include age (OR = 0.902, 95% CI: 0.869–0.937, *p* < .001), weight (OR = 0.480, 95% CI: 0.269–0.856, *p* = .013), prematurity (OR = 2.793, 95% CI: 1.069–7.294, *p* = .036), surgery time more than 60 min (OR = 3.743, 95% CI: 1.797–7.797, *p* < .001), prewarming (OR = 0.081, 95% CI: 0.038–0.173, *p* < .001), receiving fluid more than 20 mL/kg (OR = 2.938, 95% CI: 1.414–6.016, *p* = .004), and emergency surgery (OR = 2.142, 95% CI: 1.131–4.059, *p* = .019). Similar to neonates, age (OR = 0.991, 95% CI: 0.987–0.996, *p* < .001), weight (OR = 0.783, 95% CI: 0.637–0.961, *p* = .019), surgery time >60 min (OR = 2.140, 95% CI: 1.144–4.404, *p* = .017) prewarming (OR = 0.017, 95% CI: 0.008–0.037, *p* < .001), and receiving fluid >20 mL/L (OR = 3.074, 95% CI: 1.626–5.812, *p* = .001) were relevant factors to intraoperative hypothermia in infants along with the ASA grade (OR = 4.135, 95% CI: 2.602–6.572, *p* < .001).

**Table 4:** Risk factors associated with intraoperative hypothermia in neonates and infants.

Variable	OR	95 CI	*p*-Value
**Neonates**			
Age	0.902	0.869–0.937	<.001
Weight	0.480	0.269–0.856	.013
Prematurity	2.793	1.069–7.294	.036
Surgery time >60 min	3.743	1.797–7.797	<.001
Prewarming	0.081	0.038–0.173	<.001
Fluid >20 mL/kg	2.938	1.414–6.016	.004
Emergency surgery	2.142	1.131–4.059	.019
**Infants**			
Age	0.991	0.987–0.996	<.001
Weight	0.783	0.637–0.961	.019
Surgery time >60 min	2.140	1.144–4.404	.017
Prewarming	0.017	0.008–0.037	<.001
Fluid >20 mL/L	3.074	1.626–5.812	.001
ASA classification	4.135	2.602–6.537	<.001

ASA, American Society of Aneshesiologists physical status classification system.

## Discussion

This retrospective study with more than 1,000 neonates and infants receiving general anesthesia revealed a high incidence of intraoperative hypothermia (82.83% in neonates and 38.31% in infants), even when an active warming device was used in most patients. Most cases were of mild hypothermia (35°C–35.9°C), which still prolonged PACU stay, ICU stay, postoperative mechanical ventilation time, and postoperative hospital stay and increased postoperative ICU admission. In addition, intraoperative hypothermia was also associated with a high occurrence of surgical site infection, postoperative bleeding, and blood transfusion, which may delay recovery after surgeries. The risk factors of intraoperative hypothermia were largely identical but with minor differences between neonates and infants. Younger age, lower weight, longer surgery time, received more fluid, and no prewarming management were common risk factors for intraoperative hypothermia in both neonates and infants, while prematurity was a risk factor in neonates.

Hypothermia is broadly defined as a core temperature below 36.0°C in children (16, 17), and a few studies defined it as <35°C (14, 18). It has been suggested that a body temperature below 36.5°C be considered hypothermia in younger patients (16). In this study, 36°C was used to define intraoperative hypothermia since it is more commonly used. The incidence of intraoperative hypothermia was reported to range from 20% to 90% depending on the type of surgery and study population (13–16). Preterm infants and newborns were the most susceptible population to intraoperative hypothermia, with a high occurrence (19). The incidence of hypothermia in neonates in our study was 82.83%, more than double in infants, which is similarly reported previously (13, 15).

Compared with adults and older children, neonates and infants can easily have intraoperative hypothermia because of a relatively larger surface/body weight ratio, thinner keratine layer and subcutaneous fat, and an immature thermoregulatory system (20, 21). The heat loss of a naked newborn at birth in an environmental temperature of 23°C equals the heat loss of an unclothed adult at 0°C (22). Small and sick infants have a narrow thermoneutral range and are prone to thermal instability (19, 22). That is why the incidence of intraoperative hypothermia was extremely high in neonates.

Prematurity was an independent risk factor for intraoperative hypothermia in neonate patients, which may be due to their lower body weight and immature thermoregulatory system (12, 16, 20). In neonates, heat generation depends on nonshivering thermogenesis and can be initiated through a neuroendocrine pathway triggered by increased sympathetic activity and thyroid-stimulating hormone, triiodothyronine, and thyroxin release as well as norepinephrine in the brown adipose tissue (11, 23). These finally upregulate the thermogenin protein in the brown adipose tissue with a major increase around the 32nd week of gestation (24). Further, preterm infants have poor vasomotor control at birth and do not have peripheral vasoconstriction to preserve heat (11). Other risk factors for intraoperative hypothermia in neonates and infants such as younger age, lower weight, longer surgery time, and received more fluid were in accordance with other reports (7, 25, 26).

The adverse events of hypothermia are enormous such as thermal discomfort, alterations in pharmacokinetic and pharmacodynamic parameters (essentially muscle relaxants and opiates) (27), disruption of platelet function, coagulation (4, 28) and blood loss, cardiocirculatory and respiratory complications, wound healing delay, and surgical site infections (29). In neonates and preterm infants, hypothermia can lead to pulmonary hypertension, tissue hypoxia, arterial hypotension and hypoperfusion of vital organs, metabolic acidosis, and hypoglycemia (30). All these may subsequently induce multiple pathophysiological changes such as catecholaminergic response, vasoconstriction, increased metabolism, and decreased surfactant synthesis (16, 31, 32). Intraoperative hypothermia was associated with a longer postoperative extubation time, prolonged PACU, ICU, and hospital stay, and a higher rate of postoperative bleeding and transfusion, as shown clearly in our study.

Intraoperative core temperature is commonly monitored with an esophageal or nasopharyngeal temperature probe by inserting the probe into the lower third of the esophagus or nasopharynx cavity (33, 34). Other methods of core temperature measurement were tympanic membrane temperature or rectal temperature measurement (16, 35). In this study, the esophageal or nasopharyngeal temperature as the core temperature was measured. Other studies reported that only 70%–80% of pediatric patients received temperature motoring, and half of them developed hypothermia (13, 32). Improved intraoperative body temperature management, including monitoring, prevention, and warming therapy, should be formulated and implemented.

Forced-air warming and infusion warming were the most common active warming measures during the surgery. Infants often rewarm faster than adults or older children through a forced-air warmer because of the larger body surface/body weight ratio (36). Many pediatric anesthesiologists prefer forced-air warming to infusion warming because the continuous heat loss from the maintenance of infusion can usually be offset by forced-air warming (37, 38). As prevention, active prewarming is the most effective way to prevent hypothermia, which should start before anesthesia induction and last unti back to the inpatient ward or ICU or even longer during recovery. Preterm infants and neonates should be transported in a warmed incubator, and if possible it should be considered to perform surgery in the NICU for high-risk patients (20). The interruption of prewarming also can result in hypothermia (39), and with an adequate prewarming protocol, hypothermia incidence can be reduced to 20% (10), even in preterm infants (40). Our study also showed that prewarming before induction of anesthesia could protect both infants and neonates suffering from intraoperative hypothermia, indicating that the prewarming management strategy should be implemented during the whole course of anesthesia and surgery or even during postsurgery recovery.

## Conclusion

Our study indicated that although core body temperature was continuously monitored and the warming system was actively used during surgery, intraoperative hypothermia still occurred in our young patients. The detrimental effects of intraoperative hypothermia reported herein are very alarming to pediatric anaesthesiologists and surgeons for taking any necessary measures to tackle hypothermia incidence. This study also calls for routine prewarming to protect neonates and infants from hypothermia. In addition, this study is retrospective, and the patients were from a single center; hence, further studies including how to improve anesthesia management in reducing hypothermia incidence in young patients are needed.

## Data Availability

The original contributions presented in the study are included in the article/Supplementary Material; further inquiries can be directed to the corresponding authors.
